# A new definition for global bioethics: COVID-19, a case study

**DOI:** 10.1080/11287462.2021.2011001

**Published:** 2022-02-04

**Authors:** Ruth Macklin

**Affiliations:** Department of Epidemiology & Population Health (Biomedical & Bioethics Research Training), Albert Einstein College of Medicine, Bronx, NY, USA

**Keywords:** Global bioethics, COVID-19 pandemic, COVAX, cooperation among countries

## Abstract

A truly global bioethics involves cooperation and collaboration among countries. Most of the articles published in bioethics journals address a problem that exists in one or more countries, but the articles typically do not discuss solutions that require collaboration or cooperation. COVAX is one example of proposed international cooperation related to the current COVID-19. pandemic. Yet it is evident that nations have been proceeding on their own with little, if any collaboration. Despite international research ethics guidance from the World Health Organization (WHO), an article published under WHO auspices violates an ethical principle rejecting “double standards” in the conduct of global research. The COVID pandemic provides an opportunity for countries to learn from the recent lack of international cooperation and employ a multi-national strategy in future global health crises.

The call for papers for the relaunch of the journal, *Global Bioethics,* says that papers “will attempt to re-define global bioethics and deepen or broaden understandings of what “global bioethics” is and does.” Without being critical of this challenge, I wondered just what is missing in the current understanding of global bioethics or the many published articles that address such topics in bioethics journals. One thing that comes to mind is that only recently has the field dealt with critical concerns of climate change, pollution of the air and water, and how these developments have led to huge migrations, people living in squalor in migrant camps, lacking proper sanitation and nutrition. But coverage of these topics is now increasing in bioethics journals, albeit without the frequency their urgency demands.

With my curiosity piqued, I turned to a book on my shelf entitled “Global Bioethics” with the subtitle “issues of conscience for the twenty-first century.” (Green et al., 2008). The book contains essays by prominent authors in the field. Although most of the contributors are from the U.S., the volume includes authors from Canada, the Netherlands, the U.K., Switzerland, and Tanzania. The titles and subtitles are: population-level bioethics (mapping a new agenda); evolving norms of medical ethics; public health prevention research; global justice, human rights, and health; harnessing advanced technologies for global health; embryos and the stem cell debate; global norms in bioethics; and physician-assisted death (“not just for rich countries”). As the titles and topics of the papers suggest, many of the authors sought to break new ground in the field.

The journal, *Developing World Bioethics*, was launched two decades ago. Many, if not most of the authors of articles published in this journal come from low and middle-income countries (LMICs). For the most part, authors from these countries were scarce before the turn of this century, but with this journal and others, such as the *Indian Journal of Medical Ethics, BMC Medical Ethics*, and *The Journal of Bioethical Inquiry*, the number and variety of articles and authors have increased. As the topics and their authors in bioethics journals began to encompass formerly neglected parts of the world, the label “global bioethics” gained currency. Why should we now attempt to “redefine global bioethics” and “deepen or broaden understandings of what global bioethics is and does”? As I examined the table of contents of these journals and read or re-read some of the articles, one thing became apparent. Although the articles cover a variety of topics, many if not most, focus on a bioethical issue in a single country or region (for example, sub-Saharan Africa). While it is true that the countries discussed in the articles span the globe, only a minority of the articles deal with relations between and among countries. That gave me a clue to what is needed for understanding what global bioethics is and does.

## The COVID-19 pandemic

Looking for possible illustrations, my thoughts landed on the current global pandemic. Key features of recent and current events, as well as publications in scientific journals and articles in the press, highlight several key bioethical concerns. Long-standing debates on the ethics of research sponsored by rich countries and carried out in poor countries have resurfaced. Prominent among these is the question whether it is ethically acceptable to conduct placebo-controlled trials in countries that lack access to medical interventions when a successful product is already available and in use in rich countries. This debate was prominent during the AIDS epidemic when preventive medications were available in the U.S. and Europe but not in most LMICs. The return of “double standards” in international research on preventive vaccines for COVID-19 proposed in an article authored by a WHO expert group gives rise to charges of exploitation of LMICs by rich and powerful nations.

Another illustrative issue in the COVID-19 pandemic is a distinct lack of global cooperation. A rational response to a global threat would be a globally coordinated response. Yet nations have not only failed to coordinate their efforts. Some countries blame other countries for spreading COVID-19 across their borders. Even friendly neighboring countries have adopted radically different responses. One example is that of Sweden and Norway. The Swedish-Norwegian border is Europe's longest. Sweden's COVID-19 deaths per capita more than 10 times higher than in Norway. Yet in November 2020 Norway tightened its coronavirus rules and extended border controls for another six months. Norway recalled its Home Guard forces to patrol its land border with Sweden (Ahlander & Pollard, 2020). Some countries have sealed their borders to prevent travelers entering from a neighboring country with a high rate of infections, while another nearby country allows access to all.

Several developments can only be described as unethical. Countries that have a surplus of effective vaccines continue to refuse to share them with countries that currently lack access. The United States is one example, and even member countries of the European Union are exhibiting what has come to be called “vaccine nationalism.” The failure of countries to honor the cooperative arrangement known as COVAX with the World Health Organization (WHO) is a breach of their previously stated endorsement of that plan. The introduction of vaccine certificates or “passports” has been subject to the criticism that it is exclusionary and unjustly limits the freedom of movement. The recent accounts of fraud in creating such passports may even cross a legal boundary. And some critics contend that the Chinese government is hiding important information about the origins of COVID-19 in their refusal to open all records to WHO scientific investigating teams. I elaborate on these points in what follows.

## Double standards redux

A recent special issue of the *Indian Journal of Medical Ethics* (IJME) focused on an article published in the *New England Journal of Medicine* (NEJM).^1^ The article in NEJM (WHO Ad Hoc Expert Group, 2021) contends that new vaccine research using placebo controls should be carried out in countries that lack access to COVID-19 vaccines already approved for use in other countries. It says that “while it is still feasible and ethical,” ongoing vaccine trials should use “directly randomized comparisons against placebo” to collect high-quality information. The purpose is “to obtain pivotal data to improve regulatory and public health decision making,” including reliable information on longer-term safety and duration of protection (WHO Ad Hoc Expert Group, p. e2(1)). The rationale is that current vaccines are “still investigational … (under Emergency Use Listing … or similar regulatory mechanisms)” (WHO Ad Hoc Expert Group, p. e2(2)). This refers to the status of the vaccines authorized for use but not yet licensed by drug regulatory authorities in the U.S., the U.K, the European Union, and other countries. Yet millions of doses of these vaccines were already being deployed around the world, including in many middle-income countries and some low-income countries (Ghana is one example). Calling the vaccines “investigational” is technically correct since they have not been fully licensed. But it is misleading because ongoing data is not being collected from these millions of vaccinated individuals throughout the world. The article proposed to do so for participants still in current placebo-controlled phase III trials and those who will enroll in future phase III vaccine trials. But that brings us back to the fundamental questions: Are future placebo-controlled COVID-19 vaccine trials ethically permissible even before such vaccines are fully licensed? As for participants in ongoing phase III placebo-controlled trials, they should be informed that they may be eligible for a vaccine that has been approved under an emergency authorization if they choose to leave the trial in which they are enrolled.

The authors, a group of experts appointed by WHO, call for “firm commitments to maintaining blinded follow-up of participants in ongoing or future placebo-controlled trials until a licensed vaccine is fully deployed in the population” (WHO Ad Hoc Expert Group, p. e2(2)). That effectively means that participants cannot find out whether they were in the group that received the vaccines or in the placebo group even after the study formally ends: “ … we believe that trial sponsors are not ethically obligated to unblind treatment assignments for participants who desire to obtain a different investigational vaccine” (WHO Ad Hoc Expert Group, p. e2(1)). This provision effectively prevents past vaccine trial participants who received placebos from obtaining information that would enable them to protect themselves (and potentially other people) by getting one of the other conditionally approved vaccines. What is involved in “firm commitments to maintaining blinded follow-up of participants” until a licensed vaccine is available in the population? This suggestion borders on a violation of the ethical requirement that research participants may terminate their participation at any time. Strictly speaking, it doesn't violate the rule because presumably, participants can leave the trial but still not be told whether they were in the placebo group or the group that received the vaccine. But in that case, what would be the point of not disclosing that information?

Among the authors comprising the WHO Ad Hoc Expert Group, 9 are from the U.S., 3 from the U.K., 1 from Finland, 1 from India, 1 from Mexico, 1 from Jamaica, 2 from South Africa, and 3 are employes of WHO. While the imbalance in the number of authors from wealthy countries sponsoring vaccine research appears striking, the authors say they reached consensus on their analysis and conclusions. Why is the composition of the Ad Hoc Expert Group relevant? For one thing, well over half the authors are from high-income countries. Given the history of research conducted over the years in LMICs under circumstances that have been termed exploitative, it is to be expected that the World Health Organization would seek an appropriate balance of membership on an Ad Hoc group of experts called upon to give advice in the pandemic. Even more problematic than the preponderance of members from high-income countries is the fact that nine are from the U.S. Nothing in the U.S. Federal Regulations governing research with human beings addresses the use of placebos in research. Former heads of the National Institutes of Health (NIH) and the Centers for Disease Control and Prevention (CDC) defended the use of placebo controls in HIV research conducted in developing countries back in the 1990s.

Authoritative sources provide guidance in controversies regarding ethics in research with human beings. Two such international documents are in widespread use throughout the world. The first of these is the Declaration of Helsinki (DoH), issued by the World Medical Association (WMA, 2013). The second authoritative source for international research ethics is a set of guidelines prepared by the Council for International Organizations of Medical Sciences (CIOMS), a non-governmental organization based in Geneva, Switzerland (CIOMS, 2016). Both documents contain paragraphs describing the circumstances in which the use of placebo controls in research are unethical. The CIOMS publication cites WHO as a collaborator in drafting its guidelines. The cover and title page say: “Prepared by the Council for International Organizations of Medical sciences (CIOMS) in collaboration with the World Health Organization (WHO).” WHO's website contains this statement regarding the governance of its Ethics Review Committee (ERC): “The ERC is guided in its work by the World Medical Association Declaration of Helsinki (1964) last updated in 2013 as well as the *International Ethical Guidelines for Biomedical Research Involving Human Subjects*” (WHO REC). These acknowledgments clearly imply that the WHO has an obligation to adhere to the spirit and letter of the DoH and the CIOMS guidelines. The article published in NEJM, under the authorship of a WHO Ad Hoc Expert Group, violates the World Health Organization's own stated ethical commitment to adhere to these two international guidelines for research with human beings.

What emerges from this analysis of an article, published in one of the world's most prestigious medical journals, is the continued dominance of the global research enterprise by the world's richest countries during a world-wide pandemic. This is not surprising. What is surprising is the apparent acceptance of this situation by an “expert group” of authors writing under the auspices of the World Health Organization. The WHO is one of the few global organizations in a position to respect and promote global bioethics.

## COVAX: a global initiative

COVAX (the COVID-19 Vaccine Global Access initiative) is a promising global arrangement that promises an international collaboration among nations in the attempt to provide access to COVID-19 vaccines to all countries, regardless of income. COVAX is jointly led by three organizations: Gavi, the Vaccine Alliance; CEPI, the Coalition for Epidemic Preparedness Innovations, and the WHO. The aim of this alliance is to coordinate international resources to manage the purchase, supply, and allocation of the vaccines (GAVI, 2020). About 190 countries are involved in this initiative, in which the high-income countries contribute to access to vaccines for LMICs. The goal of COVAX is to provide 2 billion doses of the vaccine by the end of 2021. Despite their stated commitment to this process, several high-income countries began early bilateral negotiations with manufacturers to ensure an adequate supply for themselves. This soon led to bilateral negotiations on the part of some middle-income countries. These developments gave rise to the phrase “vaccine nationalism,” a process in which rich countries bid against one another for contracts with the manufacturers, thereby obtaining sufficient supplies for their own citizens. According to one analysis, high-income countries managed to reserve more than half of the world's 2019 COVID-19 vaccine doses, although they represent only 14% of the world's population (Kuehn, 2021). These developments show that what began as a good-faith effort to allocate vaccines in an equitable manner quickly gave way to self-interested behavior on the part of rich and powerful nations.

At the 148th session of the WHO Executive Board on January 18, 2021, the Director-General of WHO, Tedros Adhanom Ghebreyesus had this to say about equitable access to COVID-19 vaccines

I need to be blunt: the world is on the brink of a catastrophic moral failure – and the price of this failure will be paid with lives and livelihoods in the world's poorest countries. Even as they speak the language of equitable access, some countries and companies continue to prioritize bilateral deals, going around COVAX, driving up prices and attempting to jump to the front of the queue. This is wrong. Forty-four bilateral deals were signed last year, and at least 12 have already been signed this year. The situation is compounded by the fact that most manufacturers have prioritized regulatory approval in rich countries where the profits are highest, rather than submitting full dossiers to WHO.

(Ghebreyesus, 2021).

It is too soon to say whether these actions on the part of high-income (and a few middle-income) countries are undermining the hoped-for effectiveness of the COVAX initiative. But what it reveals is the difficulty of forging successful global alliances that aim to help the least advantaged countries. Unlike unilateral charitable efforts on the part of wealthy countries, a global alliance is meant to obtain a commitment on the part of those countries that signed on. It is reasonable to conclude that when some countries that signed on to COVAX began to see other countries pursuing their national interests, they saw no alternative but to follow suit. The question for global bioethics is whether persistent self-interested behavior on the part of nation states can give way to the exigencies of global cooperation and collaboration.

## Vaccine certificates and passports

The rollout of preventive anti-COVID vaccines has given rise to a set of controversies within and among countries. The controversies center on these questions: should the vaccines be mandatory for certain employes, such as healthcare or food-service workers? Even if such vaccines are not mandated by a governmental authority, may employers require a certificate confirming the vaccination of their employes? Large numbers of people in many countries are opposed to mandatory COVID-19 vaccinations on grounds that it restricts their freedom of choice. Defenders of mandatory requirements appeal to a utilitarian argument, citing the health benefits to all from a vaccinated population. These debates will have to be resolved in each country or even within states and provinces within the country. But what about entry to other countries? Globalization of the world economy now requires air, land, and sea travel across numerous national borders. What about the workers on the ships, planes, railroads, and trucks who must cross these borders in carrying out their jobs? May their employers require them to have a vaccination passport? Should international travelers be required to have a vaccination passport? May the airlines require such a certificate even if the international countries involved in the passengers’ travel do not? The focus of these questions here is limited to the global situation.

A long-standing precedent exists in the requirement of proof of a yellow fever vaccination for entry into some countries. Several countries in Africa have this requirement: Côte d’Ivoire, Uganda, Cameroon, Democratic Republic of Congo, Angola, and others. An International Certificate of Vaccination, such as WHO's “yellow card,” certifies that the traveler has received the vaccination. The current proposals for COVID-19 vaccination passports have ignited controversies that were rare, if they occurred at all, in the case of yellow fever. For one thing, at the end of March 2021, coronavirus vaccines remained in short supply or were not available at all in some countries. A requirement for a vaccine passport for international travel or to cross international borders would therefore be discriminatory for the foreseeable future. In addition, some countries may decide to accept such passports only for vaccines that have been approved within their borders (Popescu & Phelan, 2021). An example is China, a country that has said its vaccine passport will allow foreigners to enter only if they have received a Chinese vaccine (Davidson, 2021).

This is the type of situation that calls for global collaboration and cooperation. In early April 2021, WHO said it did not support mandatory proof of vaccination for international travel based on equity concerns (Stolberg & Liptak, 2021 pp. A1 and A5). An argument based on equity has a clear ethical justification: requiring a vaccination passport for individuals from countries in which vaccination rollouts are slow and access is limited is simply unjust. A different appeal to ethical considerations is the claim by opponents of certificates that requiring proof of vaccination violates individuals’ “right to medical privacy.” But like other individual rights, this one has ethically justifiable exceptions. For example, in many jurisdictions syphilis cases must be reported to a public health agency so it can find and treat anyone who has been exposed. An example that has no clear ethical justification is China's requirement that individuals must have been vaccinated with a Chinese vaccine in order to enter the country. Governments can readily make rules that apply to residents of their own countries but such rules may not be acceptable to foreigners. The likelihood that countries will come to any agreement on this matter appears to be vanishingly small.

In this age of global access to social media, it will not be long before fake vaccination certificates are available world-wide. This scam began in the U.S. only a month after the Centers for Disease Control and Prevention (CDC) introduced vaccination cards to individuals who had been vaccinated. Counterfeit cards are available on Facebook, Twitter, eBay, Shopify and Etsy. According to one state official, “We’re seeing a huge market for these false cards online” (Frenkel, 2021, pp. B1 and B5). In addition to the creation of fraudulent cards, authentic CDC cards have been stolen by some pharmacists from their workplace and then sold. This fraudulent scheme is out in the open in the U.S., with some anti-vaccine groups boasting online about getting the cards. Falsifying the CDC cards in the U.S. violates federal copyright laws, and according to one official, the sale of counterfeit and stolen cards probably breaks civil and consumer protection laws, as well (Frenkel). The U.S. Federal Bureau of Investigation (FBI) issued a Public Service Announcement on March 30, 2021, which says: “If you make or buy a fake COVID-19 vaccination record card, you endanger yourself and those around you, and you are breaking the law” (FBI, 2021).

If fake vaccine passports are widely available in the U.S. and the practice is disseminated on Facebook and other social media, fraudsters in other countries cannot be far behind. Although they will not be using copies or stolen versions of the CDC vaccination cards, other paper copies and digital versions are likely to proliferate once this catches on. It is not clear what kind of international monitoring or collaborative actions can or should be taken. But if COVID-19 vaccine certificates and passports are an ethically desirable way to help stem the ongoing tide of infections, it requires a coordinated global response.

## Investigating the origins of the covid pandemic

A final example of global bioethics that emerges from the current pandemic lies in the quest for the origin of the coronavirus SARS-CoV-2. This is a scientific endeavor, not a political one. Yet in today's hyper-partisan world, blame for the pandemic itself has been laid at the door of China, where the first evidence of COVID-19 emerged at the end of 2019. In the first year of the pandemic, the U.S. took the lead among countries in blaming China, with former president Donald Trump's continued reference to the coronavirus as “the Chinese virus.” Mike Pompeo, the former U.S. Secretary of State, used the term “Wuhan virus.” The search for the origins of the virus is not merely an effort to provide an accurate historical record. It is a critically important step in understanding and preparing for future global pandemics. To that end, the WHO organized a joint international and Chinese mission to embark on the investigation. The WHO issued its report on March 30, 2021, calling for further studies (WHO Home/News, 2021).

Soon after the report was released, another independent group of scientists criticized the effort and called for further action. The letter from this group argued that critical records and biological samples needed for a thorough investigation remained inaccessible in China. The problem was that the international group organized by WHO “had no power or mandate to act independently of their Chinese colleagues … .[E]very word in the report had to be approved by both the Chinese and the international group” (Gorman, 2021). A point of contention was whether the virus might have arisen from a laboratory incident in Wuhan, China, where the first signs of the pandemic appeared. The task of the WHO mission did not include an investigation of security or procedures at the Wuhan laboratory. One infectious disease expert in the U.S. said that China wants “to create reasonable doubt that the virus started in China” (Gorman, 2021). The Director General of the WHO, Dr. Tedros Adhanom Ghebreyesus, was quoted as saying that “a laboratory leak is the least likely hypothesis,” it “requires further investigation, potentially with additional missions … .” (Gorman, 2021).

Key features of this example illustrate the nature of a truly global bioethics. WHO, the world's leading public health organization, is a key player. The joint international team of scientists that issued the above-noted report comprised 17 Chinese and 17 international experts from 10 other countries and the WHO (WHO, Home/News, 2021). As noted above, a different group of independent scientists criticized some elements in the WHO report. The head of WHO acknowledged the need for further study. Yet the limited power of an international organization like WHO to force an individual country (China in this example) to open its laboratories for inspection demonstrates the barriers to full global cooperation.

## What global bioethics is and does

A truly global bioethics explores the ethical aspects of relations between and among nations or regions of the world. While events and circumstances within a country pertaining to ethics in research with human beings, clinical medicine, public health, and climate change are worthy of study, they do not fit under a new definition of “global bioethics”. That definition encompasses the study of cooperation and collaboration (and lack thereof) between and among nations. It embraces questions about the role of international organizations such as WHO and the influence they may exert over actions or policies of individual nations. The case study of the COVID-19 pandemic explored here provides illustrative examples.

A basic element that emerges for global bioethics is the use of diplomacy. Just as diplomacy is required for interactions among nations and member organizations of the United Nations (WHO, the World Trade Organization, UNICEF, and others), it is a necessary ingredient in global bioethics. In its series on epidemic ethics, the Ethox Center of Oxford University hosted a virtual seminar entitled “Vaccine Diplomacy During the COVID-19 Pandemic” on April 12, 2021 (Vaccine Diplomacy, 2021).^2^ Speakers at the seminar criticized vaccine nationalism and called for “vaccine cosmopolitanism.” One participant pointed out that Russia and China have taken the lead in distributing vaccines to countries that lack them. The speaker questioned whether this is altruism or rather, a self-interested way of obtaining some dominance over the countries to which it has provided vaccines. Another speaker agreed, asking whether bilateral arrangements are an example of “good” vaccine diplomacy, since they may be used to gain influence over the recipient countries. The seminar participants questioned whether countries could learn from the current flawed experience of COVID-19 vaccine rollout and use some sort of multi-national strategy the next time around. In the end, some remained skeptical about the process of international vaccine diplomacy, given the dominance of vaccine nationalism. But it does not follow that diplomacy should be ruled out in other situations that global bioethics might address and that affect much of the world, such as climate change, water and air pollution, and future pandemics.

## Notes

1 Some material in the following paragraphs is excerpted, with permission, from my contribution to the above-referenced issue of IJME: Macklin R. Double standards redux. *Indian J Med Ethics*. Published online first on March 19, 2021. DOI:

2 This and previous seminars on Epidemic Ethics are available for viewing at the website noted in the References below.
